# Association of waist circumference and BMI with premature death in young and middle-aged population

**DOI:** 10.3389/fpubh.2024.1389766

**Published:** 2024-05-30

**Authors:** Lin Hu, Xinyue Han, Miaoshuang Chen, Tao Zhang

**Affiliations:** Department of Epidemiology and Health Statistics, West China School of Public Health and West China Fourth Hospital, Sichuan University, Chengdu, China

**Keywords:** premature death, waist circumference, BMI, young and middle-aged people, inverse probability weighting, NHANES

## Abstract

**Introduction:**

Premature death is a global health indicator, significantly impacted by obesity, especially in young and middle-aged population. Both body mass index (BMI) and waist circumference (WC) assess obesity, with WC specifically indicating central obesity and showing a stronger relationship with mortality. However, despite known associations between BMI and premature death, as well as the well-recognized correlation between WC and adverse health outcomes, the specific relationship between WC and premature death remains unclear. Therefore, focusing on young and middle-aged individuals, this study aimed to reliably estimate independent and combined associations between WC, BMI and premature death, thereby providing causal evidence to support strategies for obesity management.

**Methods:**

This study involved 49,217 subjects aged 18–50 years in the United States from 1999 to 2018 National Health and Nutrition Examination Survey (NHANES). Independent and combined associations between WC and BMI with premature death across sex and age stratum were examined by Cox regression. Survey weighting and inverse probability weighting (IPW) were further considered to control selection and confounding bias. Robustness assessment has been conducted on both NHANES and China Health and Retirement Longitudinal Study (CHARLS) data.

**Results:**

A linear and positive relationship between WC and all-cause premature death was found in both males and females, with adjusted *HR*s of 1.019 (95%*CI* = 1.004–1.034) and 1.065 (95%*CI* = 1.039–1.091), respectively. Nonlinear relationships were found with respect to BMI and all-cause premature death. For females aged 36–50 with a BMI below 28.6 kg/m^2^, the risk of premature death decreased as BMI increased, indicated by adjusted *HR*s of 0.856 (95%*CI* = 0.790–0.927). Joint analysis showed among people living with obesity, a larger WC increased premature death risk (*HR* = 1.924, 95%*CI* = 1.444–2.564).

**Discussion:**

WC and BMI exhibited prominent associations with premature death in young and middle-aged population. Maintaining an appropriate WC and BMI bears significant implications for preventing premature death.

## 1 Introduction

Premature death, is defined as dying before reaching the average life expectancy. It is widely used as a measure of population health internationally (1–3), and could be used to assess the “unnecessary” or “avoidable” burden of mortality. Noncommunicable diseases (NCDs) are the primary cause of premature deaths worldwide, accounting for approximately 79% of deaths among people aged 30–70 (4–7). Significantly, obesity, a major risk factor for NCDs, is associated with premature mortality in adults (5, 8, 9), especially among young and middle-aged individuals (10, 11), indicating reducing obesity incidence is a key strategy to prevent premature death.

Nevertheless, the association between obesity and mortality substantially varies by studies with different obesity indicators (6, 12), populations and designs. Body mass index (BMI) is mostly used to define obesity and is thought to be associated with premature death (13–15). However, specifically in BMI-related literature, there’s discussion of the term “obesity paradox,” where people living with obesity or overweight may correlate with better outcomes (16–18). BMI is not an accurate body fat indicator and cannot identify sarcopenic obesity. The adverse consequences of obesity may be closely related to the amount of visceral fat, and simple generalized obesity is not sufficient to explain the risk of obesity-related comorbidities (19). Therefore, central obesity represented by waist circumference (WC) and waist-hip ratio (WHR) should also be considered, which is more related to mortality (20–22). Compared with WHR, WC could be a better tool in practice for its significant correlation with visceral fat and fewer anthropometric measurement requirements (23–25). Furthermore, WHO also suggests that although BMI and abdominal adiposity measures may be highly correlated, considering the utility of joint use of the two indicators is desirable (26). In addition, most studies evaluate the association between obesity and mortality in patients with specific diseases such as diabetes (27), cancer (28), and COVID-19 (29), rather than a nationally representative general population. In the study design, some studies use self-reported measures instead of technician-measured data to conduct analyses, which could result in an inaccurate assessment of obesity (6, 30). Additionally, the majority of effect estimation studies could not control important confounding factors, such as smoking, making it impossible to get a true effect (31, 32).

In addition to the limitations in existing research mentioned above, despite the well-recognized correlation between WC and adverse health outcomes, there is no research focusing on the pattern of association between WC and premature death, and it remains unclear whether WC is an independent risk factor in addition to BMI. Moreover, obesity is a long-term process, which runs through a person’s whole life course. The process of obesity management requires the joint management of individual-community-medical institutions. Therefore, focusing on young and middle-aged individuals, this study aimed to reliably estimate independent and combined associations between WC, BMI and premature death with controlling selection and confounding bias scientifically. By this approach, we aimed to ascertain the precise effects of BMI and WC on premature death across different subgroups, thereby providing causal evidence to support strategies for obesity management.

## 2 Methods

### 2.1 Study population

This study used 1999–2018 survey data from NHANES (33), which was a nationally representative survey conducted by the United States Centers for Disease Control and Prevention. A complex, stratified, multistage probability sampling design was applied to select representative participants of the civilian, noninstitutionalized resident population of the United States. Multiple data, such as questionnaires, physical exams, and laboratory measurements were collected, with individuals’ mortality status accessed through the National Death Index (NDI). Detailed information on the survey was described elsewhere (34). Written informed consent was provided by all participants, and the protocol was approved by the Ethics Review Board of the National Center for Health Statistics (NCHS) (35).

There were 49,217 participants aged between 18 and 50 years old (young and middle-aged people) in this study. Referring to previous literature (36–38), among these participants, individuals were excluded if they had a missing baseline WC or BMI (*n* = 3,977), had missing follow-up death outcomes (*n* = 78), or were pregnant (*n* = 1,499). In addition, to avoid reverse causation, individuals who died within 2 years of the baseline survey (*n* = 129) and had WC or BMI distributed below the 1st centile (*n* = 1,292) were further excluded. The final sample consisted of 42,242 participants.

### 2.2 Assessment of exposures and outcome

WC and BMI were the exposures of interest. WC was measured to the nearest 1 mm just above the iliac crest by using a steel tape at baseline survey, with results recorded in centimeter (cm). BMI was calculated as weight in kilograms divided by height in meters squared, rounded to one decimal place (39). The outcome was all-cause premature death, defined as death from any death caused before 70 years old (7).

### 2.3 Assessment of covariates

**Figure 1:**
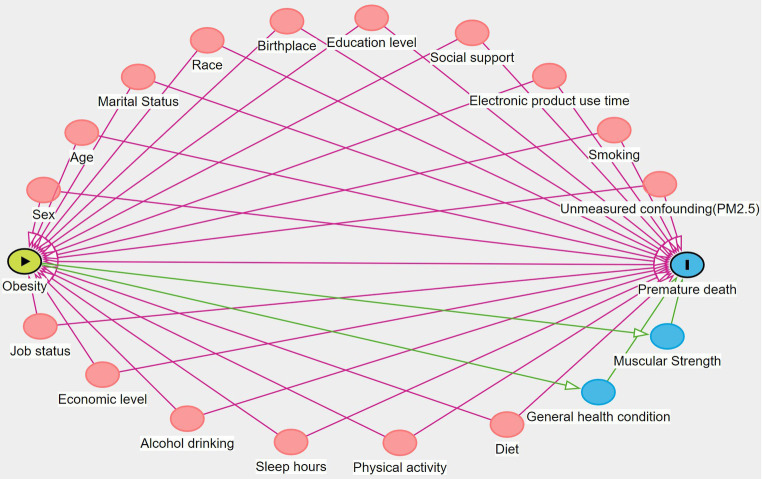
DAG for assessing confounding covariates in the relationship between obesity and premature death. The red circle represents confounding variables and the blue circle represents mediating variables.

Based on the literature review and expert knowledge, this study drew a causal directed acyclic graph (DAG) (40). In a causal DAG, the backdoor path is the non-causal path between the treatment A and the outcome Y, linking A and Y through the common cause L (Supplementary Figure S4). It is the presence of the common cause L creates an additional source of association between A and Y, which we refer to as confounding for the effect of A on Y (Supplementary file). According to this DAG and the backdoor criterion (Figure 1), confounding covariates for WC and premature death were selected, including demographics (sex, age, marital status, race, birthplace) and socioeconomic factors (education level, job status, social support and economic level), lifestyle factors (smoking, alcohol drinking, electronic product use time, sleep hours, and physical activity), and diet (number of restaurant meals and healthy eating). Among them, job status included long working hours and job type. Social support included emotional support, financial support, and the number of close friends. The economic level was represented by the family poverty level index (Family PIR). Physical activity included moderate activity and vigorous activity. In addition, WC and BMI were mutually adjusted to assess their independent effects on premature death. Covariates such as hypertension and diabetes were not included as confounding covariates because they are on the causal pathway of obesity and premature death. Including them in the model may lead to incorrect adjustment and introduce bias (6, 24, 37, 41). As a result, a total of 21 confounding covariates were incorporated into the model, and their definitions are shown in Supplementary Table S1.

### 2.4 Statistical methods

#### 2.4.1 Participants characteristics study

Analysis of this study was conducted according to the official requirements (42) of NHANES and considered the complex survey design of the cohort. When describing the baseline characteristics of the respondents, categorical variables were summarized as number (percentage), and continuous variables as *M* (*P*_25_, *P*_75_). The ***χ***^**2**^ test and rank sum test were, respectively, used for categorical variables and continuous variables to test whether there was a difference between the no premature death group and the premature death group.

#### 2.4.2 Independent effect analysis

To investigate the independent association patterns between exposures and premature death by different sexes, analyses were conducted separately for WC and BMI. We calculated person-months from baseline to the date of death, or 31 December 2019, whichever came first. The proportional hazard assumption (PH assumption) was assessed using weighted Schoenfeld residuals (43). A survey-weighted stratified Cox regression was constructed by stratifying variables that failed to meet the PH assumption. We tested and revealed the nonlinearity by modeling exposures using classical restricted cubic splines with 4 knots (located at the 10th, 30th, 70th, and 90th percentiles). The reference value was the median (44, 45). In cases of nonlinearity, a recursive algorithm was utilized to ascertain the inflection point, signifying a pivotal shift in the relationship between exposures and premature mortality. Piecewise fitting was performed on both sides of the inflection point (46). If the results did not present non-linearity, the survey-weighted stratified Cox regression was constructed in a linear form. Results were presented as changes in the hazard ratio (*HR*) for a 1-cm increment in WC and a 1-kg/m^2^ increment in BMI.

Inverse probability weighting was further used in this study to control for confounding bias. Since the exposure was a continuous variable, a generalized propensity score model was first constructed (47, 48), with the exposure as the dependent variable and the confounding covariates as independent variables. To account for survey weights, we also incorporated them as covariates, following suggestions from the literature (49). Then inverse probability weights were calculated by estimating the probability of the exposure using a normal distribution. The balance test was performed by calculating the average absolute correlation between exposure and confounding covariates to ensure balance across confounding covariates, and all average absolute correlations of inverse probability weights in this study were smaller than 0.1, indicating balance across confounding covariates (Supplementary Figures S1, S2). In the second step, a stratified Cox proportional hazards regression model was constructed to study the association between exposures and premature death. Inverse probability weights and survey weights were considered at the same time. This study totally adjusted for 21 covariates selected by DAG. Referring to previous literature (50, 51), missing values were imputed via multiple imputation methods.

Moreover, referencing previous studies (52–54), we also did subgroup analyses to explore whether the association of exposures and premature death varied across age (18–35 years old and 36–50 years old), stratified by sex.

#### 2.4.3 Joint analysis

To examine the combined effects of WC and BMI, we studied the association between WC and premature death within the BMI category (24). Because of the high correlation between BMI and WC, it was difficult to model the impact of WC on premature mortality across all categories of BMI (55). Therefore, participants were stratified by BMI category based on WHO standards (normal weight “18.5 to 24.9 kg/m^2^,” overweight “25 to 29.9 kg/m^2^,” and obesity “> 30 kg/m^2^”). The WC category was based on the sex-specific median cutting points, which was the median sex-specific WC of the population in each BMI category. In the joint analysis, owing to considering inverse probability weighting with binary exposure, a balance test was performed by calculating the Standardized Mean Difference (*SMD*) to ensure balance across confounding covariates (Supplementary Figure S3).

#### 2.4.4 Robustness assessment

To assess the possible impact of unmeasured confounding, the classical *E*-value was calculated (56). The *E*-value is defined as the minimum strength of association on the risk ratio scale that an unmeasured confounder would need to have with both the treatment and the outcome to fully explain away a specific treatment-outcome association, conditional on the measured covariates (Supplementary file). The model without considering the inverse probability weights was also constructed. In addition, the joint analysis of this study was repeated in the China Health and Retirement Longitudinal Study (CHARLS) data (57).

All analyses were performed with R 4.0.5, using the package ‘survey’ and ‘rms’ to account for survey design and restricted cubic spline fitting (58), respectively. Self-written functions were used to determine inflection points. In all tests, a two-tailed *p* < 0.05 was considered statistically significant.

## 3 Results

### 3.1 Study population characteristics

There were 42,242 young and middle-aged participants in the study, with a median age of 34 years at the time of this study participation and 21,277 were males (50.37%). The survey-weighted median WC and BMI were 93.30 (*IQR* = 83.30–104.40) cm and 26.97 (*IQR* = 23.43–31.38) kg/m^2^, respectively. Over a median follow-up of 140 (*IQR* = 100–156) months, a total of 1,005 all-cause premature deaths were observed. The baseline characteristics were presented in Supplementary Table S2.

Comparisons of the premature death group with the no premature death group showed significant differences in sex, age, race, birthplace, education level, family PIR, smoking, sleep hours, and WC (all *P* < 0.05). In general, young and middle-aged people who died prematurely were more likely to be males, 36–50 years old, non-Hispanic white, born domestically (in the 50 US states or Washington of DC), having a high school degree or less, having a family poverty index >1.85, smoking every day, sleeping 6 h or less, and with larger WC.

### 3.2 Association of WC and premature death

**Figure 2:**
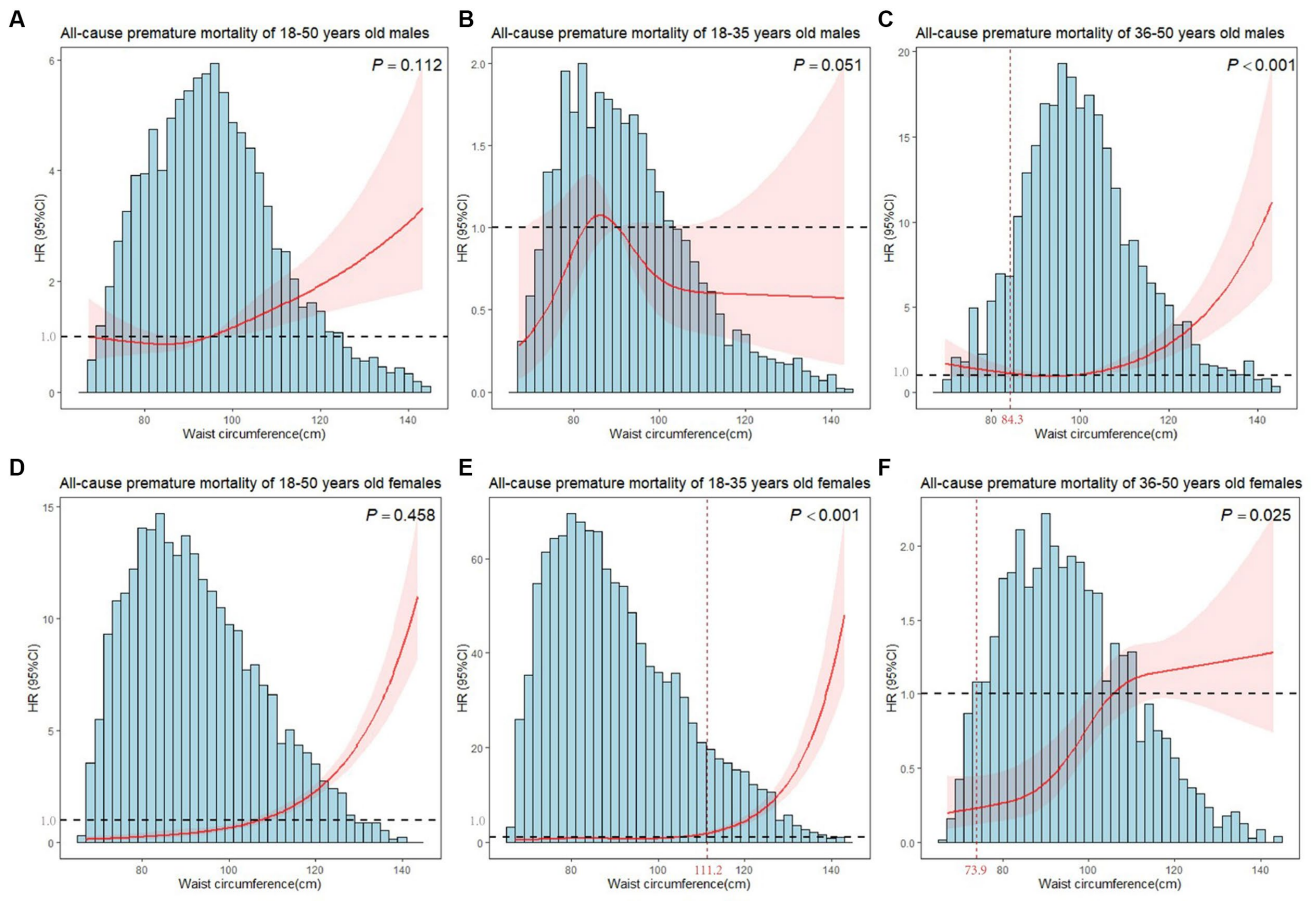
Plots of all-cause premature mortality *HR* from multivariate adjusted stratified Cox regression analysis using restricted cubic splines of WC with 4 degrees of freedom. **(A)** Model for 18–50 years old males. **(B)** Model for 18–35 years old males. **(C)** Model for 36–50 years old males. **(D)** Model for 18–50 years old females. **(E)** Model for 18–35 years old females. **(F)** Model for 36–50 years old females. *P* showed the test for nonlinearity. **(B,C,E,F)** models were adjusted for marital status, race, birthplace, education level, long working hours, job type, emotional support, financial support, number of close friends, economic level, smoking, alcohol drinking, electronic product use time, sleep hours, moderate activity, vigorous activity, number of restaurant meals, healthy eating, BMI. **(A,D)** models additionally adjusted for age. The horizontal line in the graph indicated a premature death *HR* of 1, and the vertical line marked the inflection point.

In Figure 2, restricted cubic splines were used to flexibly model and visualize the relationship of WC with premature mortality, and linear associations were observed in males and females (*P*_nonlinear_ = 0.112, *P*_nonlinear_ = 0.458, respectively). Hence, WC was included in the models in a linear form, with statistically significant results (*P* < 0.01). The risk of premature death increased with WC gain in males and females, with adjusted *HR*s of 1.019 (1.004–1.034) and 1.065 (1.039–1.091), respectively (Table 1).

**Table 1:** Sex-specific threshold effect analysis of WC on premature death by age groups.

	**Age group**	**Inflection point**	**Adjusted *HR* below Inflection point (95%*CI*)**	***P*-value**	**Adjusted *HR* above Inflection point (95%*CI*)**	***P*-value**	**likelihood ratio test**
**Males**							
	**18 < =Age < =35**	-^a^	0.997 (0.972-1.023)	0.832	…^b^	…^b^	
	**36 < =Age < =50**	84.3	0.904 (0.845–0.966)	**0.003**	1.052 (1.033–1.072)	**<0.001**	<0.001
	**Overall**	-^a^	1.019 (1.004-1.034)	**0.011**	…^b^	…^b^	
**Females**							
	**18 < =Age < =35**	111.2	1.041 (0.996–1.088)	0.072	1.134 (1.089–1.180)	**<0.001**	<0.001
	**36 < =Age < =50**	73.9	0.899 (0.794–1.018)	0.094	1.029 (1.012–1.046)	**<0.001**	<0.001
	**Overall**	-^a^	1.065 (1.039-1.091)	**<0.001**	…^b^	…^b^	

Bold values indicates results that are statistically significant. ^a^Not available for linear relationship. Piecewise fitting was used to construct models. ^b^Same as the adjusted *HR* below inflection point. The models were adjusted for marital status, race, birthplace, education level, long working hours, job type, emotional support, financial support, number of close friends, economic level, smoking, alcohol drinking, electronic product use time, sleep hours, moderate activity, vigorous activity, number of restaurant meals, healthy eating, BMI.

The age-specific analysis presented nonlinear associations between WC and premature mortality for 36–50 years old males, 18–35 years old females and 36–50 years old females (all *P*_nonlinear_ < 0.001). We estimated the premature mortality risk of the 36–50 years old males to reach the inflection point at WC of 84.3 cm, with inverse associations below (*HR* = 0.904, 95%*CI* = 0.845–0.966), and positive associations above (*HR* = 1.052, 95%*CI* = 1.033–1.072). For 36–50 years old females, a marked increase in risk at the middle WC, but minimal elevation in risk at lower and higher WC was observed (Figure 2). WC was a significant predictor of all-cause premature mortality in the 18–35 years old and 36–50 years old female subgroups when WCs were larger than inflection points (111.2 cm and 73.9 cm, respectively). However, no significant results (*P* > 0.05) were found in females when WCs were smaller than inflection points. Moreover, WC did not affect premature death in 18–35 years old males (Table 1).

### 3.3 Association of BMI and premature death

**Figure 3:**
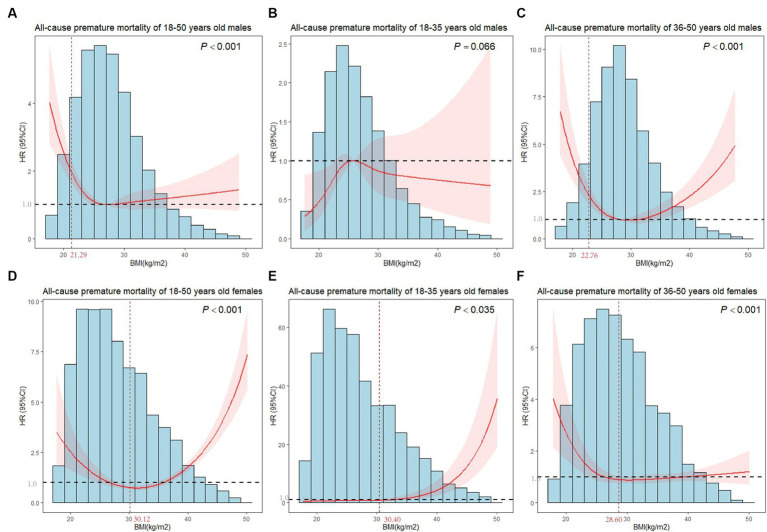
Plots of all-cause premature mortality *HR* from multivariate adjusted stratified Cox regression analysis using restricted cubic splines of BMI with 4 degrees of freedom. **(A)** Model for 18–50 years old males. **(B)** Model for 18–35 years old males. **(C)** Model for 36–50 years old males. **(D)** Model for 18–50 years old females. **(E)** Model for 18–35 years old females. **(F)** Model for 36–50 years old females. **(B,C,E,F)** models were adjusted for marital status, race, birthplace, education level, long working hours, job type, emotional support, financial support, number of close friends, economic level, smoking, alcohol drinking, electronic product use time, sleep hours, moderate activity, vigorous activity, number of restaurant meals, healthy eating, BMI. **(A,D)** models additionally adjusted for age. The horizontal line in the graph indicated a premature death *HR* of 1, and the vertical line marked the inflection point.

Figure 3 showed nonlinear associations of BMI with premature mortality in both males and females (all *P*_nonlinear_ < 0.001). The inflection point of males was 21.29 kg/m^2^, below which a significant inverse relationship was observed (*HR* = 0.562, 95%*CI* = 0.421–0.752). In females, a reduction of the risk within the lower range of BMI was found (*HR* = 0.854, 95%*CI* = 0.796–0.916) which reached the lowest risk around 30.12 kg/m^2^ and increased thereafter (*HR* = 1.132, 95%*CI* = 1.069–1.199, Table 2).

**Table 2:** Sex-specific threshold effect analysis of BMI on premature death by age groups.

	**Age group**	**Inflection point**	**Adjusted *HR* below Inflection point (95%*CI*)**	***P*-value**	**Adjusted *HR* above Inflection point (95%*CI*)**	***P*-value**	**likelihood ratio test**
**Males**							
	**18 < =Age < =35**	-^a^	1.021(0.980-1.063)	0.325	…^b^	…^b^	
	**36 < =Age < =50**	22.76	0.597(0.485–0.736)	**<0.001**	1.069(1.022–1.118)	**0.004**	<0.001
	**Overall**	21.29	0.562(0.421–0.752)	**<0.001**	1.010(0.982–1.039)	0.495	<0.001
**Females**							
	**18 < =Age < =35**	30.4	0.990(0.828–1.184)	0.913	1.227(1.156–1.302)	**<0.001**	<0.001
	**36 < =Age < =50**	28.6	0.856(0.790–0.927)	**<0.001**	1.024(0.987–1.063)	0.208	<0.001
	**Overall**	30.12	0.854(0.796–0.916)	**<0.001**	1.132(1.069–1.199)	**<0.001**	<0.05

Bold values indicates results that are statistically significant. ^a^Not available for a linear relationship. Piecewise fitting was used to construct models. ^b^Same as before. ^a^The BMI classification was based on the WHO standard. ^b^Same as the adjusted HR below inflection point. The models were adjusted for marital status, race, birthplace, education level, long working hours, job type, emotional support, financial support, number of close friends, economic level, smoking, alcohol drinking, electronic product use time, sleep hours, moderate activity, vigorous activity, number of restaurant meals, healthy eating, WC.

After age-stratified, we observed nonlinear associations between BMI and premature mortality in 36–50 years old males, 18–35 years old females and 36–50 years old females (*P*_nonlinear_ < 0.005). As shown in Table 2, the inflection point for all-cause mortality risk among 36–50 years old males was at a BMI of 22.76 kg/m^2^, with inverse associations below (*HR* = 0.597, 95%*CI* = 0.485–0.736), and positive associations above (*HR* = 1.069, 95%*CI* = 1.022–1.118). The inflection point for the effect of BMI of 18–35 years old females on premature death was 30.40 kg/m^2^. When the BMI of females was larger than 30.40 kg/m^2^, the risk of premature death increased with an increment in BMI (*HR* = 1.227, 95%*CI* = 1.156–1.302). In 36–50 years old females, we found a substantial reduction in risk below BMI of 28.60 kg/m^2^ (*HR* = 0.856, 95%*CI* = 0.790–0.927), whereas a nonsignificant positive association was observed above 28.60 kg/m^2^. Similarly, BMI did not affect premature death in 18–35 years old males.

### 3.4 Joint analysis

We also determined the combined association of WC and BMI with premature death. Table 3 showed that in the group with normal weight or overweight, WC larger than the median did not increase the risk of premature death (*HR* = 1.346, 95%*CI* = 0.937–1.934; *HR* = 1.136, 95%*CI* = 0.863–1.495). In the group with obesity, the premature death risk of individuals with WC larger than the median was 1.92 times of individuals with WC smaller than the median (*HR* = 1.924, 95%*CI* = 1.444–2.564).

**Table 3:** Joint Analysis of BMI and WC on premature death in young and middle-aged people.

**BMI category** ^**a**^	**WC category** ^**b**^	**Adjusted *HR* (95%*CI*)**	***P*-value**	***E-value*** ^**c**^
**Normal weight**				
	Males >81.7, Females >78.8	1.346(0.937–1.934)	0.107	2.03(1.00)
**Overweight**				
	Males >95.6, Females >91.0	1.136(0.863–1.495)	0.362	1.53(1.00)
**Obesity**				
	Males >111.4, Females >107.9	1.924(1.444–2.564)	**<0.001**	3.26(2.24)

Bold values indicates results that are statistically significant. ^a^The BMI classification was based on the WHO standard. ^b^The WC category was based on the sex-specific median cutting points. ^c^*E*-value for point estimates and the lower bound of the 95%*CI* of the *HR*.The models were adjusted for sex, age, marital status, race, birthplace, education level, long working hours, job type, emotional support, financial support, number of close friends, economic level, smoking, alcohol drinking, electronic product use time, sleep hours, moderate activity, vigorous activity, number of restaurant meals, healthy eating.

### 3.5 Robustness assessment

The *E*-values of *HR* values obtained from different models were calculated for men and women, respectively. Results indicated that only the *HR* of unmeasured confounding exceeded the corresponding *E*-value would change the relationship between WC, BMI, and premature death found in this study (Tables 3, 4). However, since this study had adjusted for a sufficient number of confounding covariates, the likelihood of including unmeasured confounding with this strength to distort the findings was relatively low. Besides, the survey-weighted model without considering inverse probability weight showed similar results, demonstrating the robustness of the study. In addition, consistent results were observed based on the joint effect of CHARLS, particularly among individuals who are overweight, with an adjusted *HR* of 1.245 (95% *CI* = 0.614–2.528).

**Table 4:** *HRs* and *E*-values for different genders and age groups.

	**Age group**	**WC**			**BMI**		
		**Inflection point**	***HR* (95%*CI*)**	***E*-value** ^**b**^	**Inflection point**	***HR* (95%*CI*)**	***E*-value** ^**b**^
**Males**							
	**18 < =Age < =35**	-^a^	0.997(0.972-1.023)	1.06(1.00)	-^a^	1.021(0.980-1.063)	1.17(1.00)
	**36 < =Age < =50**	<84.3	0.904(0.845–0.966)	1.45(1.23)	<22.76	0.597(0.485–0.736)	2.74(2.06)
		>84.3	1.052(1.033–1.072)	1.29(1.22)	>22.76	1.069(1.022–1.118)	1.34(1.17)
	**Overall**	-^a^	1.019(1.004-1.034)	1.16(1.07)	<21.29	0.562(0.421–0.752)	2.96(1.99)
					>21.29	1.010(0.982–1.039)	1.11(1.00)
**Females**							
	**18 < =Age < =35**	<111.2	1.041(0.996–1.088)	1.25(1.00)	<30.40	0.990(0.828–1.184)	1.11(1.00)
		>111.2	1.134(1.089–1.180)	1.52(1.40)	>30.40	1.227(1.156–1.302)	1.75(1.58)
	**36 < =Age < =50**	<73.9	0.899(0.794–1.018)	1.47(1.00)	<28.60	0.856(0.790–0.927)	1.61(1.37)
		>73.9	1.029(1.012–1.046)	1.20(1.12)	>28.60	1.024(0.987–1.063)	1.18(1.00)
	**Overall**	-^a^	1.065(1.039-1.091)	1.33(1.24)	<30.12	0.854(0.796–0.916)	1.62(1.41)
					>30.12	1.132(1.069–1.199)	1.52(1.34)

Bold values indicates results that are statistically significant. ^a^Not available for linear relationship. ^b^*E*-value for point estimates and the lower bound of the 95%*CI* of the *HR*.

## 4 Discussion

In this prospective study involving 49,217 young and middle-aged Americans, with controlling selection and confounding bias scientifically, we first found a linear and positive relationship between WC and all-cause premature death in young and middle-aged people, and nonlinear relationships were found with respect to BMI and all-cause premature death. The results varied slightly across subgroups defined by gender and age. Nonlinear relationships between WC and all-cause premature death were found in males aged 36–50 years old and in females aged 18–35 years old and 36–50 years old. Additionally, we also discovered the combined effect of BMI and WC, with a larger WC being a risk factor for premature death among individuals classified with obesity based on BMI category.

Although the outcome of most studies was death rather than premature death, they still supported the results of this study (6). A pooled analysis of 650,000 adults from 11 prospective cohort studies also found a positive linear association between men and women. The association between WC and premature mortality may be due to the fact that a larger WC is highly correlated with visceral fat and is a reflection of visceral fat accumulation. Inflammation of visceral adipose tissue mediates metabolic disturbances and is associated with an increased risk of cancer, cardiovascular disease, and even death (36, 37, 59, 60).

When the BMI was low, a protective effect was found, consistent with other literature (61). This may be because there is an adverse effect on premature death when BMI is below the lower limit of normal weight. Lower BMI is related to reduced muscle mass, which leads to lower mobility. In addition, a study has also pointed out a protective effect of moderately increased body fat (15). Subcutaneous fat provides additional metabolic reserves, which are needed in stressful situations when the activation of the neurohormonal system and inflammation require additional energy. However, people with low BMI have less subcutaneous fat and are unable to provide extra energy to cope with stressful situations (62, 63).

We also found that for females with a BMI below 30.12 kg/m^2^, who are living with overweight or obesity, the risk of premature death decreased with the increase in BMI, suggesting the existence of an obesity paradox (64). Through further subgroup analysis, it was found that the obesity paradox appeared in 36–50 years old females living with overweight, similar to other studies (61, 65, 66). Some studies have proposed that age was a confounding factor leading to the obesity paradox. With the increase of age, fat loss becomes slower, and nutrition status is relatively better, so obesity becomes a protective factor (67). It may also be related to the distribution of body composition. Young and middle-aged people have more fat distributed in the limbs, larger muscle mass, and relatively less visceral fat, which is conducive to the prognosis of the disease (68). Other possible reasons are better compliance and higher rates of treatment after an illness among them (65). Furthermore, the influence of risk factors in young and middle-aged people is relatively small. Similar to the study by Vest et al., the phenomenon of the obesity paradox in women, while not present in men, may be due to differences in the distribution of fat in the sexes (69–71). Men tend to store excessive fat in visceral fat deposits, whereas women usually store fat in peripheral subcutaneous distributions. Excessive visceral fat could increase the risk of developing metabolic syndrome and cardiovascular diseases, whereas femoral-gluteal fat might be beneficial as a “sink” for lipids (72).

Neither WC nor BMI was associated with premature death among men aged 18–35 in this study. Previous literature has mentioned that men aged 18–35 are often highly active physically (73, 74), which could decrease the risk of premature death associated with obesity. Therefore, the results of this study were that obesity had little effect on premature death among men aged 18–35 (75). Despite this, men aged 18–35 should still avoid obesity, as neglecting to do so could increase the risk of premature death as they age. While this study accounted for moderate and vigorous physical activities as confounding factors, they may not fully represent the actual total physical activity situation. For example, daily walking was not included, so the confounding effect caused by physical activity in men aged 18–35 cannot be completely controlled.

Studies supporting the obesity paradox utilized BMI for defining obesity, which did not discriminate between lean mass and fatmass (76). Therefore, many studies have recommended the use of WC as a better means of identifying obesity (77). Geum Joon Cho et al. also reported that WC should be considered in the assessment of obesity-related health risks (14). However, for the strong association between BMI and death, BMI was still broadly used as an indicator of obesity in practical work. Considering the unique characteristics of BMI and WC, combining BMI and WC could better improve the ability to identify the risk of premature death (6, 78). Some studies have reported findings similar to this research, indicating increased mortality in adults living with obesity (79). The visceral microenvironment may be intrinsically toxic to arterial health, and WC is highly correlated with harmful visceral abdominal tissue (80–83). People who have larger WC and are living with obesity have higher amounts of visceral fat mass, which increases the risk of cardiometabolic diseases and is positively and significantly associated with a higher risk of all-cause mortality (84, 85). Therefore, we also recommend that the combined assessment of WC and BMI was conducive to identifying high-risk groups of premature death among people living with obesity more effectively.

We observed that young and middle-aged individuals, at a critical stage in the life course where the incidence of obesity and adverse outcomes is increasing, had both waist circumference (WC) and body mass index (BMI) associated with premature death. Based on our findings, we suggested that young and middle-aged people should avoid abdominal obesity and maintain a suitable BMI, rather than striving for a low BMI. Furthermore, the impact of obesity on the human body runs through the whole life course. Young and middle-aged people can establish a strong foundation for their health in later life by prioritizing this issue now. In addition to the individual’s own emphasis on obesity, communities and hospitals also play an important role in this process. There are often slogans in the community to remind residents to avoid obesity, increase exercise activities, and pay attention to a healthy diet (86–88). Medical service institutions and related societies may offer lifestyle guidelines for people living with obesity at various stages, while medical and social organizations can provide weight management programs (89–91).

This study used a large and nationally representative sample of the population, with long-term follow-up of death outcomes to ensure the quality of research data. Further, with in-depth consideration of the study population, a comprehensive selection of confounding covariates was performed to investigate the relationships between WC and BMI with premature death. The results of the inverse probability weighted balance assessment showed that confounding factors distributed balanced by inverse probability weighting and survey weighting, which enhanced the interpretability and validity of our analysis to provide evidence for effective research. Robustness assessment also suggested robustness of the association between exposures and premature death in young and middle-aged people. However, this study also has some limitations. First, WC and BMI were measured at baseline only, so we were unable to assess the effect of exposure change on premature death in the follow-up assessment. Second, due to the limitation of sample size, this study was also unable to study the specific causes of premature death. Third, due to data limitations, we cannot study the relationship between WHR and premature death. Fourthly, although NHANES employed standardized operating procedures and measurements of anthropometric indices like WC by trained technicians, with rigorous quality control measures implemented (24, 92, 93), there were slight differences in measurement standards compared to the WHO (94). While this limitation was not expected to affect the effect estimation of this study, which was the main aim of the study. Considering the possible influence on the calculation of inflection points, problems such as measurement methods are worth further study in the future. Nevertheless, the results of this study with causal inference methods controlling for possible selection bias and confounding bias, still could provide non-negligible support for further research on the obesity paradox and causal mechanism in the future. In the next studies, the pattern of association between WHR and premature death, WC and BMI with different causes of premature death will also be further explored, and possible mechanisms underlying the different patterns of associations will be explored in greater depth.

In conclusion, based on the results of this study, we concluded that WC and BMI exhibited prominent associations with all-cause premature death. WC and BMI were risk factors for the occurrence of premature death in young and middle-aged adults, except for males with small BMI and females with BMI below obesity. For young and middle-aged people living with obesity, regardless of gender, there is a need to control the growth of WC, and thus reduce the risk of premature death. The clinical importance of maintaining an appropriate WC and BMI bears significant implications for preventing premature mortality attributable to obesity.

## Data availability statement

Publicly available datasets were analyzed in this study. This data can be found here: US National Health and Nutrition Examination Survey (NHANES): https://www.cdc.gov/nchs/nhanes/index.htm; China Health and Retirement Longitudinal Study (CHARLS): https://charls.pku.edu.cn/.

## Ethics statement

All methods were carried out in accordance with the Data Security Law of the People’s Republic of China, the World Medical Association's Declaration of Helsinki, and other relevant guidelines and regulations. All the data involved in this research were obtained from NHANES and CHARLS. NHANES was approved by the Ethics Review Board of the National Center for Health Statistics. CHARLS was approved by the Ethics Review Committee of Peking University.

## Author contributions

LH: Supervision, Writing – original draft, Writing – review & editing, Conceptualization, Data curation, Formal analysis, Investigation, Methodology, Project administration, Resources, Software, Validation, Visualization. XH: Data curation, Investigation, Validation, Writing – review & editing. MC: Data curation, Investigation, Writing – review & editing. TZ: Writing – review & editing, Funding acquisition, Supervision, Writing – original draft.
