# Supplementary material for: Association of waist circumference and BMI with premature death in young and middle-aged population
Source: Front Public Health. 2024 May 30;12:1389766. doi: 10.3389/fpubh.2024.1389766 (PMC11169795; doi:10.3389/fpubh.2024.1389766)
Supplement: Supplementary file 1 [file Data_Sheet_1.docx]

Table 1. Variable assignment table

|  | **Variables** | **Value** |
| --- | --- | --- |
| Demographics | Sex | Male =1， Female =2 |
|  | Age | 18-35 years old =1，36-50 years old =2 |
|  | Marital Status | Married =1， Unmarried =2 |
|  | Race | Mexican American =1， Other Hispanic =2， Non-Hispanic White =3， Non-Hispanic Black =4， Other Race =5 |
|  | Birthplace | Born in 50 US States or Washington, DC =1， Other place=2 |
| Socioeconomic factors | Education level | High school degree or less =1， Above high school degree =2 |
|  | Long working hours | Yes=1，No=2 |
|  | Job type | An employee of a private company, business, or individual for wages, salary, or commission =1， A federal government employee =2， A state government employee =3， A local government employee =4， Self-employed in own business, professional practice or farm =5， Working without pay in family business or farm =6 |
|  | Emotional support | Yes=1，No=2 |
|  | Financial support | Yes=1，No=2 |
|  | Number of close friends | 0 =1，1-4 =2，≥5 =3 |
|  | Family poverty level index (Family PIR) | Family PIR ≤ 1.30=1，1.30 < Family PIR ≤ 1.85=2， Family PIR > 1.85=3 |
| Lifestyle factors | Smoking | Every day =1， Some days =2， Not at all =3 |
|  | Alcohol drinking | Yes=1，No=2 |
|  | Electronic product use time | Less than 1 hour/day =1，1-3 hours/day=2，4 hours or more/day =3 |
|  | Sleep hours | ≤6 hours =1，7-9 hours =2，≥10 hours =3 |
|  | Moderate activity | Yes=1，No=2 |
|  | Vigorous activity | Yes=1，No=2 |
| Diet | Number of restaurant meals | 0-5times/month=1，6-15 times/month =2，≥16 times/month =3 |
|  | Healthy eating | Good=1，Fair=2，Poor=3 |

Table 2. The characteristics of study population

| **Variables** | **Value** | **All young and middle-aged people (*N*=42242)** | **No premature death (*N*=41237)** | **Premature death (*N*=1005)** | **All young and middle-aged people (*N*=42242)^a^** | **No premature death (*N*=41237)^a^** | **Premature death(*N*=1005)^a^** | ***P* ^a^** |
| --- | --- | --- | --- | --- | --- | --- | --- | --- |
| **Sex** | Male | 21277(50.37) | 20659(50.1) | 618(61.49) | 21277(51.06) | 20659(50.8) | 618(62.25) | <0.001 |
|  | Female | 20965(49.63) | 20578(49.9) | 387(38.51) | 20965(48.94) | 20578(49.2) | 387(37.75) |  |
| **Age** | 18-35 years old | 23403(55.40) | 23138(56.11) | 265(26.37) | 23403(51.67) | 23138(52.32) | 265(23.76) | <0.001 |
|  | 36-50 years old | 18839(44.60) | 18099(43.89) | 740(73.63) | 18839(48.33) | 18099(47.68) | 740(76.24) |  |
| **Marital Status** | Married | 18118(46.24) | 17713(46.35) | 405(41.88) | 18118(51.27) | 17713(51.43) | 405(44.76) | 0.080 |
|  | Unmarried | 21062(53.76) | 20500(53.65) | 562(58.12) | 21062(48.73) | 20500(48.57) | 562(55.24) |  |
| **Race** | Mexican American | 8899(21.07) | 8735(21.18) | 164(16.32) | 8899(10.81) | 8735(10.9) | 164(6.97) | <0.001 |
|  | Other Hispanic | 4059(9.61) | 4015(9.74) | 44(4.38) | 4059(6.49) | 4015(6.55) | 44(3.71) |  |
|  | Non-Hispanic White | 16539(39.15) | 16096(39.03) | 443(44.08) | 16539(63.47) | 16096(63.44) | 443(64.73) |  |
|  | Non-Hispanic Black | 9233(21.86) | 8915(21.62) | 318(31.64) | 9233(12.23) | 8915(12.06) | 318(19.25) |  |
|  | Other Race | 3512(8.31) | 3476(8.43) | 36(3.58) | 3512(7.01) | 3476(7.05) | 36(5.34) |  |
| **Birthplace** | Born in 50 US States or Washington, DC | 30462(72.14) | 29603(71.81) | 859(85.47) | 30462(80.99) | 29603(80.77) | 859(90.65) | <0.001 |
|  | Other places | 11767(27.86) | 11621(28.19) | 146(14.53) | 11767(19.01) | 11621(19.23) | 146(9.35) |  |
| **Education level** | High school degree or less | 17931(47.87) | 17336(47.48) | 595(62.96) | 17931(40.39) | 17336(39.96) | 595(58.29) | <0.001 |
|  | Above high school degree | 19526(52.13) | 19176(52.52) | 350(37.04) | 19526(59.61) | 19176(60.04) | 350(41.71) |  |
| **Long working hours** | Yes | 3082(38.78) | 3016(38.57) | 66(51.16) | 3082(40.67) | 3016(40.53) | 66(49.34) | 0.240 |
|  | No | 4866(61.22) | 4803(61.43) | 63(48.84) | 4866(59.33) | 4803(59.47) | 63(50.66) |  |
| **Job type** | An employee of a private company, business, or individual for wages, salary, or commission | 24029(79.2) | 23593(79.23) | 436(77.58) | 24029(77.48) | 23593(77.5) | 436(76.15) | 0.823 |
|  | A federal government employee | 630(2.08) | 622(2.09) | 8(1.42) | 630(2.12) | 622(2.14) | 8(1.32) |  |
|  | A state government employee | 1663(5.48) | 1630(5.47) | 33(5.87) | 1663(5.66) | 1630(5.65) | 33(6.29) |  |
|  | A local government employee | 1641(5.41) | 1606(5.39) | 35(6.23) | 1641(6.27) | 1606(6.26) | 35(6.85) |  |
|  | Self-employed in own business, professional practice or farm | 2302(7.59) | 2253(7.57) | 49(8.72) | 2302(8.28) | 2253(8.27) | 49(9.01) |  |
|  | Working without pay in family business or farm | 73(0.24) | 72(0.24) | 1(0.18) | 73(0.19) | 72(0.18) | 1(0.37) |  |
| **Emotional support** | Yes | 6701(90.57) | 6371(90.46) | 330(92.7) | 6701(93.71) | 6371(93.81) | 330(91.49) | 0.504 |
|  | No | 698(9.43) | 672(9.54) | 26(7.3) | 698(6.29) | 672(6.19) | 26(8.51) |  |
| **Financial support** | Yes | 5258(71.23) | 4987(70.97) | 271(76.34) | 5258(76.75) | 4987(77) | 271(70.96) | 0.345 |
|  | No | 2124(28.77) | 2040(29.03) | 84(23.66) | 2124(23.25) | 2040(23) | 84(29.04) |  |
| **Number of close friends** | 0 | 449(6.07) | 421(5.98) | 28(7.87) | 449(3.68) | 421(3.5) | 28(7.99) | 0.120 |
|  | 1-4 | 3332(45.08) | 3167(45.01) | 165(46.35) | 3332(41.16) | 3167(40.77) | 165(50.17) |  |
|  | ≥5 | 3611(48.85) | 3448(49.01) | 163(45.79) | 3611(55.16) | 3448(55.73) | 163(41.84) |  |
| **Family PIR** | ≤1.30 | 13323(35.99) | 12907(35.73) | 416(46.48) | 13323(25.53) | 12907(25.29) | 416(35.84) | 0.006 |
|  | 1.30-≤1.85 | 5379(14.53) | 5256(14.55) | 123(13.74) | 5379(12.59) | 5256(12.6) | 123(12.26) |  |
|  | > 1.85 | 18320(49.48) | 17964(49.72) | 356(39.78) | 18320(61.88) | 17964(62.12) | 356(51.89) |  |
| **Smoking** | Every day | 8213(52.05) | 7751(51.25) | 462(70.64) | 8213(49.61) | 7751(48.71) | 462(71.6) | <0.001 |
|  | Some days | 2051(13) | 2007(13.27) | 44(6.73) | 2051(11.61) | 2007(11.87) | 44(5.33) |  |
|  | Not at all | 5515(34.95) | 5367(35.48) | 148(22.63) | 5515(38.78) | 5367(39.42) | 148(23.06) |  |
| **Alcohol drinking** | Yes | 24902(75.21) | 24210(75.16) | 692(76.72) | 24902(79.3) | 24210(79.28) | 692(80.01) | 0.791 |
|  | No | 8210(24.79) | 8000(24.84) | 210(23.28) | 8210(20.7) | 8000(20.72) | 210(19.99) |  |
| **Electronic product use time** | Less than 1 hour/day | 372(4.73) | 357(4.73) | 15(4.72) | 372(4.32) | 357(4.37) | 15(3.08) | 0.589 |
|  | 1-3 hours/day | 4823(61.38) | 4630(61.41) | 193(60.69) | 4823(65.03) | 4630(65.1) | 193(63.37) |  |
|  | 4 hours or more/day | 2663(33.89) | 2553(33.86) | 110(34.59) | 2663(30.64) | 2553(30.53) | 110(33.55) |  |
| **Sleep hours** | ≤6 hours | 12538(36.58) | 12216(36.28) | 322(53.49) | 12538(34.48) | 12216(34.14) | 322(55.04) | 0.001 |
|  | 7-9 hours | 20447(59.66) | 20183(59.94) | 264(43.85) | 20447(62.33) | 20183(62.64) | 264(43.48) |  |
|  | ≥10 hours | 1290(3.76) | 1274(3.78) | 16(2.66) | 1290(3.2) | 1274(3.23) | 16(1.48) |  |
| **Moderate activity** | Yes | 5495(25.1) | 5300(25.01) | 195(27.82) | 5495(27.24) | 5300(27.15) | 195(30.27) | 0.245 |
|  | No | 16400(74.9) | 15894(74.99) | 506(72.18) | 16400(72.76) | 15894(72.85) | 506(69.73) |  |
| **Vigorous activity** | Yes | 4656(24.35) | 4506(24.39) | 150(23.22) | 4656(24.64) | 4506(24.65) | 150(24.32) | 0.890 |
|  | No | 14467(75.65) | 13971(75.61) | 496(76.78) | 14467(75.36) | 13971(75.35) | 496(75.68) |  |
| **Number of restaurant meals** | 0-5times/month | 25922(73.4) | 25312(73.24) | 610(81.01) | 25922(71.91) | 25312(71.83) | 610(76.08) | 0.448 |
|  | 6-15 times/month | 8417(23.83) | 8286(23.97) | 131(17.4) | 8417(25.28) | 8286(25.36) | 131(21.29) |  |
|  | ≥16 times/month | 976(2.76) | 964(2.79) | 12(1.59) | 976(2.81) | 964(2.81) | 12(2.63) |  |
| **Healthy eating** | Good | 22531(65.65) | 22171(65.76) | 360(59.7) | 22531(68.65) | 22171(68.83) | 360(57.73) | 0.101 |
|  | Fair | 9401(27.39) | 9241(27.41) | 160(26.53) | 9401(24.85) | 9241(24.82) | 160(26.52) |  |
|  | Poor | 2388(6.96) | 2305(6.84) | 83(13.76) | 2388(6.5) | 2305(6.35) | 83(15.75) |  |
| **Waist circumference** |  | 93.00(82.80,104.30) | 93.0(82.7,104.2) | 96.2(86.9,107.8) | 93.30(83.30, 104.40) | 93.2(83.2,104.3) | 97.3(87.0,109.0) | 0.002 |
| **BMI** |  | 27.10(23.47,31.60) | 27.10(23.46,31.60) | 27.18(23.72,33.06) | 26.97(23.43,31.38) | 26.97(23.42,31.34) | 27.10(23.72,33.31) | 0.223 |

^a^ *P* accounted for complex survey designs.


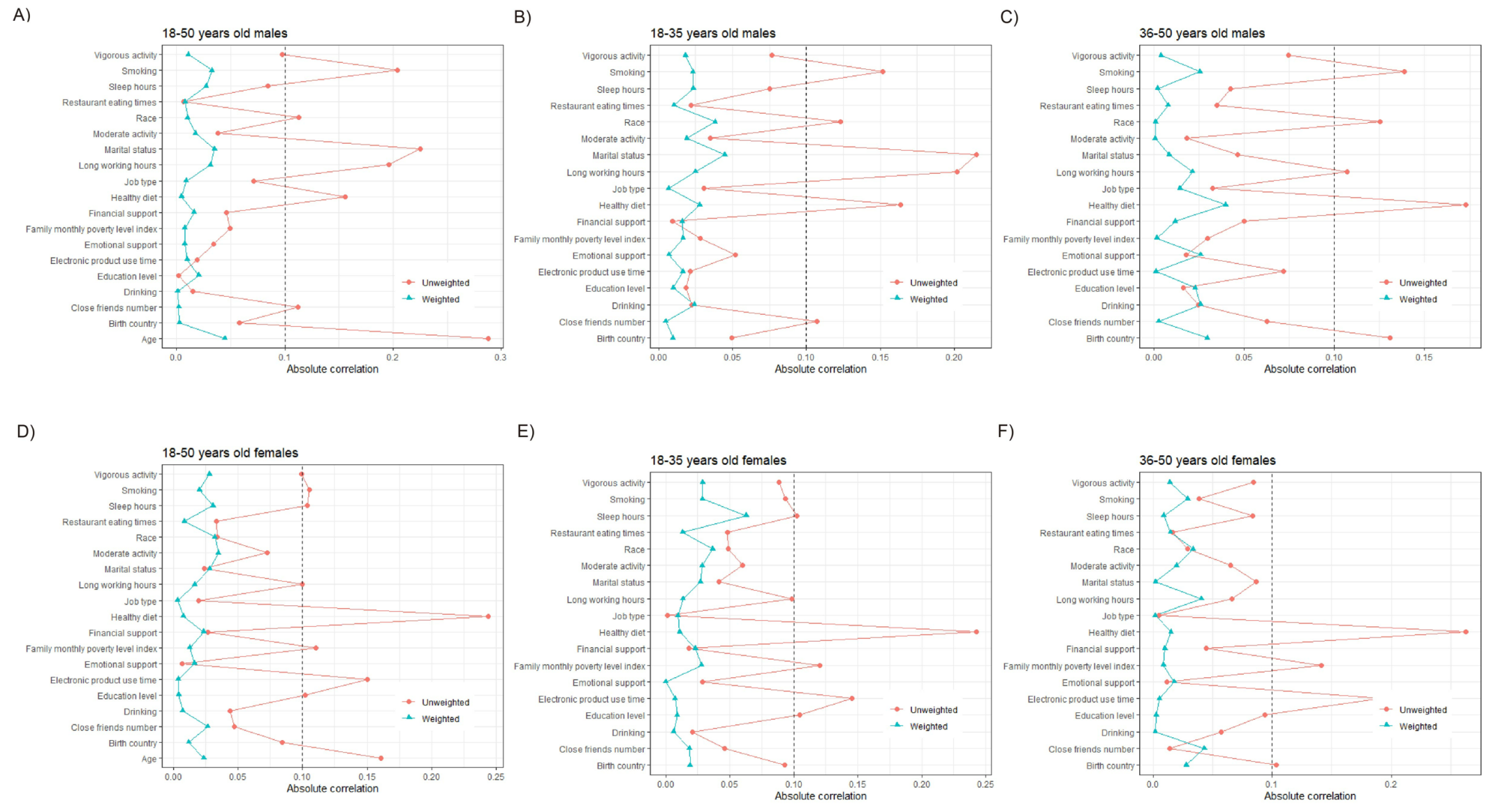


**Figure 1. Balance assessment of Confounding covariates with waist circumference as the exposure.** A) Model for 18-50 years old males. B) Model for 18-35 years old males. C) Model for 36-50 years old males. D) Model for 18-50 years old females. E) Model for 18-35 years old females. F) Model for 36-50 years old females.


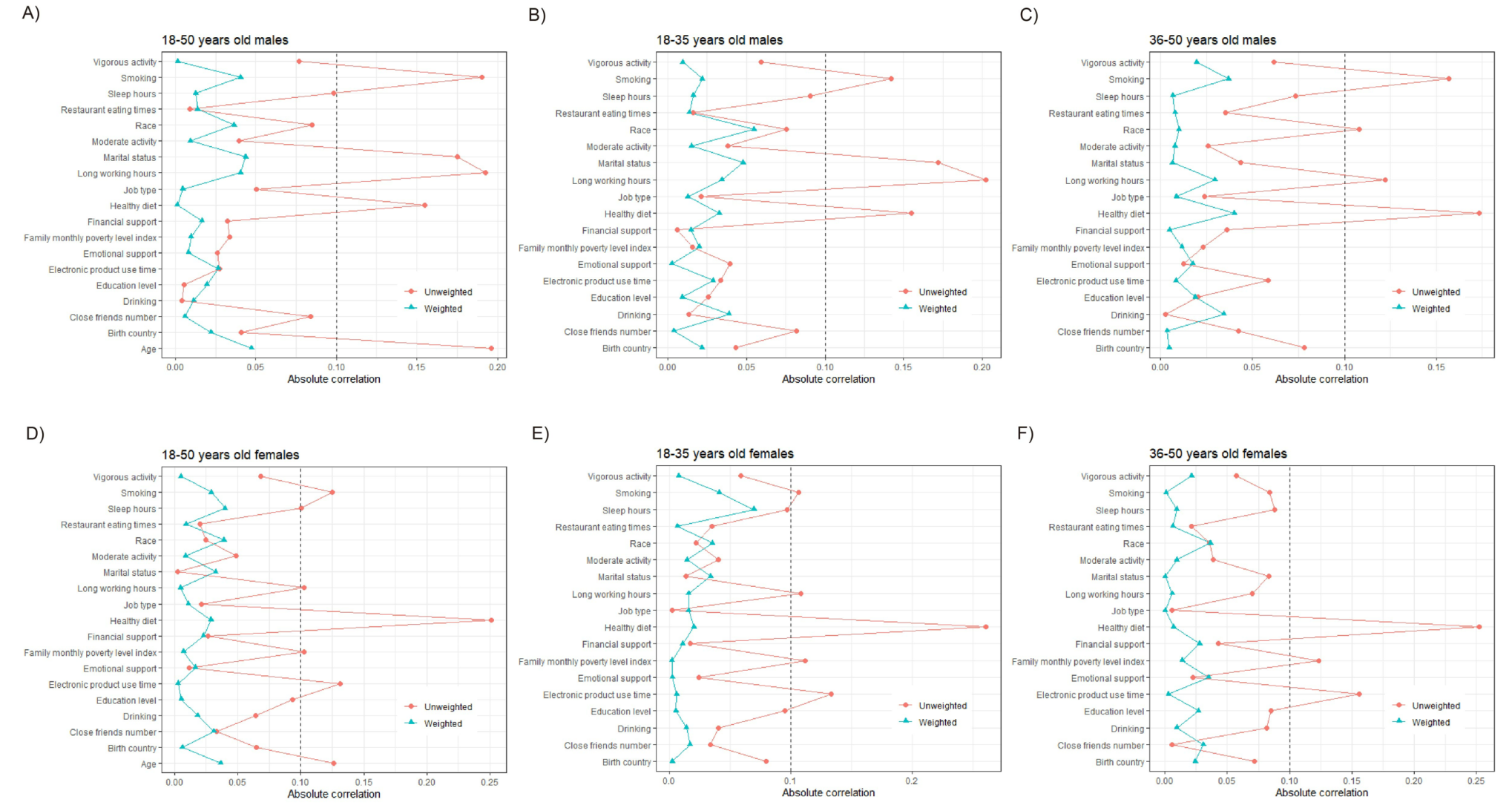


**Figure 2. Balance assessment of Confounding covariates with BMI as the exposure.** A) Model for 18-50 years old males. B) Model for 18-35 years old males. C) Model for 36-50 years old males. D) Model for 18-50 years old females. E) Model for 18-35 years old females. F) Model for 36-50 years old females.


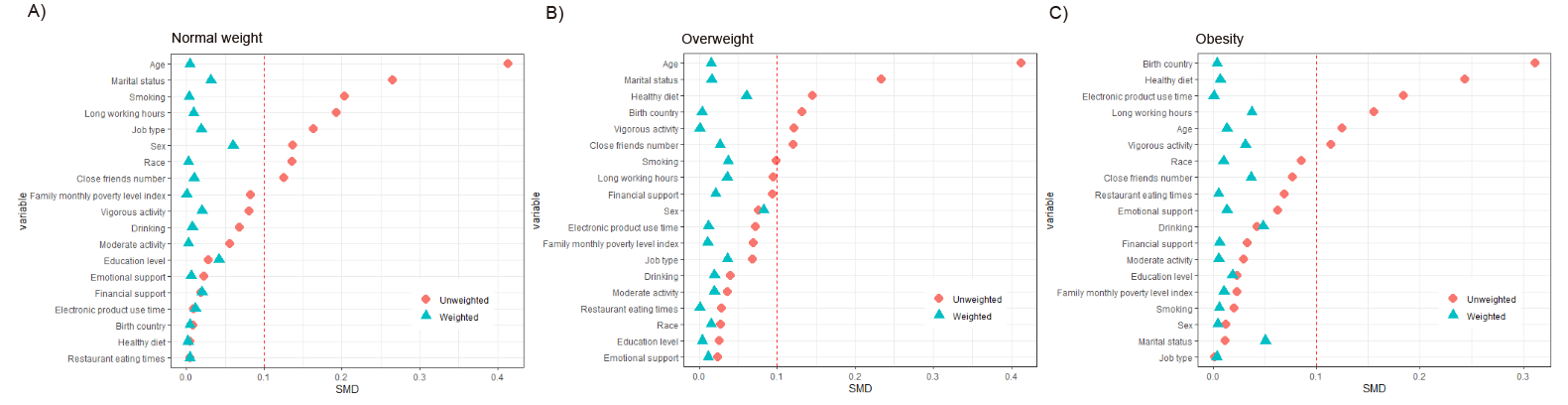


**Figure 3. Balance assessment of Confounding covariates with binary waist circumference as the exposure.** A) Model for the people with normal weight. B) Model for the people with overweight. C) Model for the people with obesity.

**Backdoor criterion**

The structure of confounding, the bias due to common causes of treatment and outcome, can be represented by using causal diagrams. For example, the diagram in Figure 4 depicts a treatment A, an outcome Y, and their shared (or common) cause L. This diagram shows two sources of association between treatment and outcome: 1) the path A → Y that represents the causal effect of A on Y, and 2) the path A ← L → Y between A and Y that includes the common cause L. The path A ← L → Y that links A and Y through their common cause L is an example of a backdoor path. In a causal DAG, a backdoor path is a noncausal path between treatment and outcome that remains even if all arrows pointing from treatment to other variables (the descendants of treatment) are removed. That is, the path has an arrow pointing into treatment.

If the common cause L did not exist in Figure 4, then the only path between treatment and outcome would be A → Y, and thus the total association between A and Y would be due to the causal effect of A on Y. But the presence of the common cause L creates an additional source of association between the treatment A and the outcome Y, which we refer to as confounding for the effect of A on Y. Because of confounding, the associational risk ratio does not equal the causal risk ratio. Therefore, association is not causation.

In Figure 4 there is confounding because the treatment A and the outcome Y share the cause L, i.e., because there is an open backdoor path between A and Y through L. However, this backdoor path can be blocked by conditioning on L. Thus, if the investigators collected data on for all individuals, there is no unmeasured confounding given (1-3).


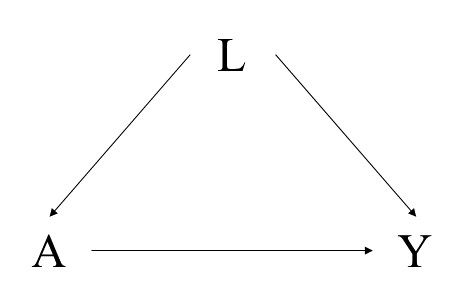


**Figure 4**

***E*-value**

The *E*-value is defined as the minimum strength of association on the risk ratio scale that an unmeasured confounder would need to have with both the treatment and the outcome to fully explain away a specific treatment-outcome association, conditional on the measured covariates. A large *E*-value implies considerable unmeasured confounding would be needed to explain away an effect estimate. A small *E*-value implies little unmeasured confounding would be needed to explain away an effect estimate (4). *E*-values can be directly interpreted as “betting scores” or “likelihood ratios”, or can be interpreted as “how likely is the rejection to be true?” (5). For the observed *HR* in this study, *E*-value was calculated as follows: $E-value=HR+\sqrt{HR*(HR-1)}$.

**Reference**

1. Hernán MA RJ. Causal Inference: What If: Boca Raton: Chapman & Hall/CRC; 2020.

2. Greenland S, Pearl J, Robins JM. Causal diagrams for epidemiologic research. Epidemiology. 1999;10(1):37-48.

3. J P. Causality. 2nd ed: Cambridge University Press; 2009.

4. VanderWeele TJ, Ding P. Sensitivity Analysis in Observational Research: Introducing the E-Value. Ann Intern Med. 2017;167(4):268-74.

5. Yang Y, Liu H, Liu Y, Zhou L, Zheng X, Yue R, et al. E-value: a superior alternative to P-value and its adjustments in DNA methylation studies. Brief Bioinform. 2023;24(4).
